# Designing neoantigen cancer vaccines, trials, and outcomes

**DOI:** 10.3389/fimmu.2023.1105420

**Published:** 2023-02-09

**Authors:** Nupur Biswas, Shweta Chakrabarti, Vijay Padul, Lawrence D. Jones, Shashaanka Ashili

**Affiliations:** ^1^ Rhenix Lifesciences, Hyderabad, India; ^2^ CureScience, San Diego, CA, United States

**Keywords:** neoantigen vaccine, cancer immunotherapy, clinical trials, WES, NGS - next generation sequencing

## Abstract

Neoantigen vaccines are based on epitopes of antigenic parts of mutant proteins expressed in cancer cells. These highly immunogenic antigens may trigger the immune system to combat cancer cells. Improvements in sequencing technology and computational tools have resulted in several clinical trials of neoantigen vaccines on cancer patients. In this review, we have looked into the design of the vaccines which are undergoing several clinical trials. We have discussed the criteria, processes, and challenges associated with the design of neoantigens. We searched different databases to track the ongoing clinical trials and their reported outcomes. We observed, in several trials, the vaccines boost the immune system to combat the cancer cells while maintaining a reasonable margin of safety. Detection of neoantigens has led to the development of several databases. Adjuvants also play a catalytic role in improving the efficacy of the vaccine. Through this review, we can conclude that the efficacy of vaccines can make it a potential treatment across different types of cancers.

## 1 Introduction

Cancer is an outcome of the abnormal proliferation of cells. The abnormal proliferation leads to the unrestricted growth of cells in the form of a tumor. If the abnormally proliferating cells invade surrounding normal tissue and/or spread all over the body, then it turns into cancer (1). Normal somatic cells turn into cancer cells due to genetic alterations. The divergent nature of genetic alterations, which mostly include mutations, has made cancer a complex disease. Several types of mutations are accumulated within the cells, starting from the embryonic state. But only a combination of mutations in multiple genes leads to cancer (2). Those mutations, translated to changes in the amino acid arrangement, create mutated proteins that are new to the body’s adaptive immune system. The mutant peptides, usually ~ 8-25 mer long peptides around the mutated sites are considered as neoantigens. According to Xia et al. a neoantigen with validated immunogenicity is termed as neoepitope and a neoantigen with uncertain immunogenicity is termed as neopeptide (3).

Broadly there are two types of tumor antigens, Tumor Associated Antigens (TAA) and Tumor-Specific Antigens (TSA) (4). Neoantigens are a subclass of TSAs and differ from TAAs. TAAs are not unique to tumor cells but neoantigens are. TAAs are derived from over-expressed proteins which may also be present in normal cells (5). Neoantigens are tumor-specific and expressed in tumor cells only. There were attempts at cancer vaccines targeting TAAs as well; however, the results were not so promising (6). Trials have also been conducted targeting differentiation antigens which appear at particular phases of cell differentiation but they can be expressed in both tumor and normal cells (7). Neoantigens arise from different types of mutations in DNA which include point mutations, insertions, deletions, gene fusions (8–10), and even frameshift mutations in genes that may or may not be oncogenes or tumor suppressor genes. As point mutations are more frequent, they are more often used as neoantigen candidates.

Neoepitopes are already present in the patient’s body but only localized in the tumor cells. In neoantigen immunotherapy, synthetically made neopeptides are administered to the patients. The goal is to stimulate the immune system to recognize the neoantigens so that CD8+ and CD4+ T cells are activated to recognize and to destroy the cancer cells. However, the success of this process depends on several factors, the foremost among them being the successful loading and presentation of the neopeptides on human leukocyte antigens (HLA) proteins. Personalized neoantigen vaccines may train the immune system to identify and kill the neopeptide-presenting cancer cells. Apart from provoking immunogenicity, other advantages of the neoantigen vaccine are that it can be given to outpatients and side effects are not significant (11). Neoantigens are patient-specific, however, few of them may be shared among multiple patients (4). Mutations are not always random, driver mutations often appear in multiple patients. It opens up the possibility of shared neoepitopes for at least in the subgroup of patients sharing common mutations (12, 13).

**Figure 1:**
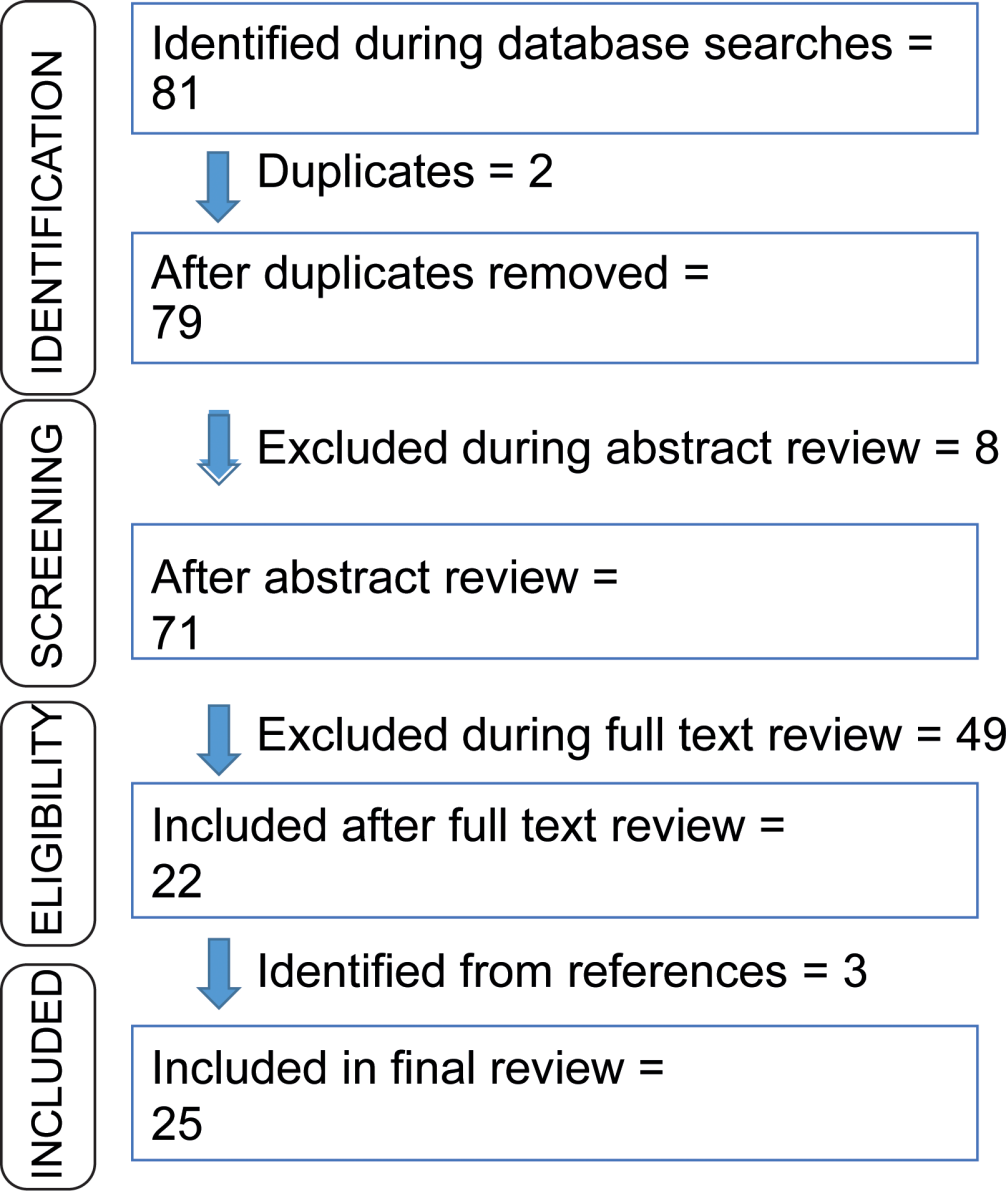
Flow chart describing the article selection process for this review.

In the last few years due to the cost-effectiveness of sequencing technologies, neoantigen vaccines have appeared as emergent immunotherapy. In this review, we are addressing the criteria, processes, and challenges associated with neoantigen vaccine design. We observed that several clinical trials are ongoing. A few research groups have also reported their trial results. Although the number of enrolled patients is less, several clinical trials are reporting encouraging results. Utilizing the national clinical trial website of NIH (https://clinicaltrials.gov/ct2/home), a search afforded 126 results using a combination of keywords ‘cancer’, ‘neoantigen vaccine’, and ‘neoepitope’, of which 39 trials were either active, terminated or completed (searched on December 20, 2022). Among them, we have discussed 26 trials in this review which involve the extraction of mutated peptides from sequence data, with the administration of them to the patients for evaluation. In order to determine the outcomes from these neoantigen vaccine therapy clinical trials, we completed a keywords search in PubMed using combinations of keywords ‘neoantigen’, ‘neoepitope’, ‘cancer’, ‘vaccine’, and ‘clinical trials’. We identified 79 articles for article type ‘clinical trial’ to date (PubMed accessed on December 19, 2022). We included clinical trials where neoantigens were administered on human subjects only. **Figure 1** shows our selection procedure for reviewing the outcomes. Based on these conditions, we summarized the ongoing clinical trials. Below we have discussed the reported outcomes in the neoantigen vaccine research.

## 2 Neoantigen design

The neoantigen design process starts with the identification of all types of somatic mutations from the whole genome or the exome sequencing of tumor samples. All mutations do not lead to effective neoantigens. For being identified as a neoepitope as well as a successful candidate for neoantigen vaccine therapy, the peptide must bind with the HLA molecules and the neoantigen-HLA complex must be able to stimulate neopeptide-specific T cells of the immune system (14). Hence, after the identification of various neopeptides, the potentially effective neopeptides are selected based on the predicted probability of neopeptide-HLA binding (15). These predictions are done using different algorithms which often use existing data of experimentally validated peptides which are available in the databases like Immune Epitope Database and Analysis Resource (IEDB) (16). Multiple combinations of algorithms are followed to identify the key parameters behind the neopeptide-HLA binding (17). Structural modeling considering spatial features has also been used to predict HLA binding energies as well as CD8+ T cell responses towards neoantigen (18, 19).

### 2.1 Criteria

As previously indicated, the primary requirement of designing a neoantigen is that the peptide must bind with the HLA molecules and the peptide-HLA complex must be able to stimulate T cells of the immune system (14). However, there are additional criteria that should be considered for effective design. These criteria include proper selection of target somatic mutations, the exclusivity of the peptide production in the cancer cells, abundant expression, processing by antigen presentation pathway, binding of the peptide fragment to host specific HLA proteins, and mutated allele frequency. All of these criteria are difficult to satisfy and a compromised or prioritized choice is made using immunoinformatics approaches (20). Researchers have developed multiple pipelines for the selection of neopeptides, such as pVACtools (21), Vaxrank (22), MuPeXI (23), TSNAD (24), and pTuneos (25). Each of these pipelines has its selection process and results in the lists of neopeptides. However, these lists very often differ from each other. In the following section, we will discuss the generalized approach required for vaccine design.

### 2.2 Processes

The personalized neopeptide vaccine design requires information on the mutations in proteins which are translated from the mutation sites of the DNA in cancer cells. This information is extracted by comparing the DNA sequences of normal and tumor cells. Either whole genome sequencing (WGS) or whole exome sequencing (WES) data of DNA of both tumor and normal cells is required. However, since of the entire genome, only exonic parts are only translated to peptides, WES is sufficient to detect somatic mutations. Moreover, compared to WGS, WES is more economical considering both clinical and computational costs. Additionally, the mutated proteins should be expressed in the tumor cells, to ensure that mRNA sequencing of tumor cell mRNAs is also performed.

**Figure 2:**
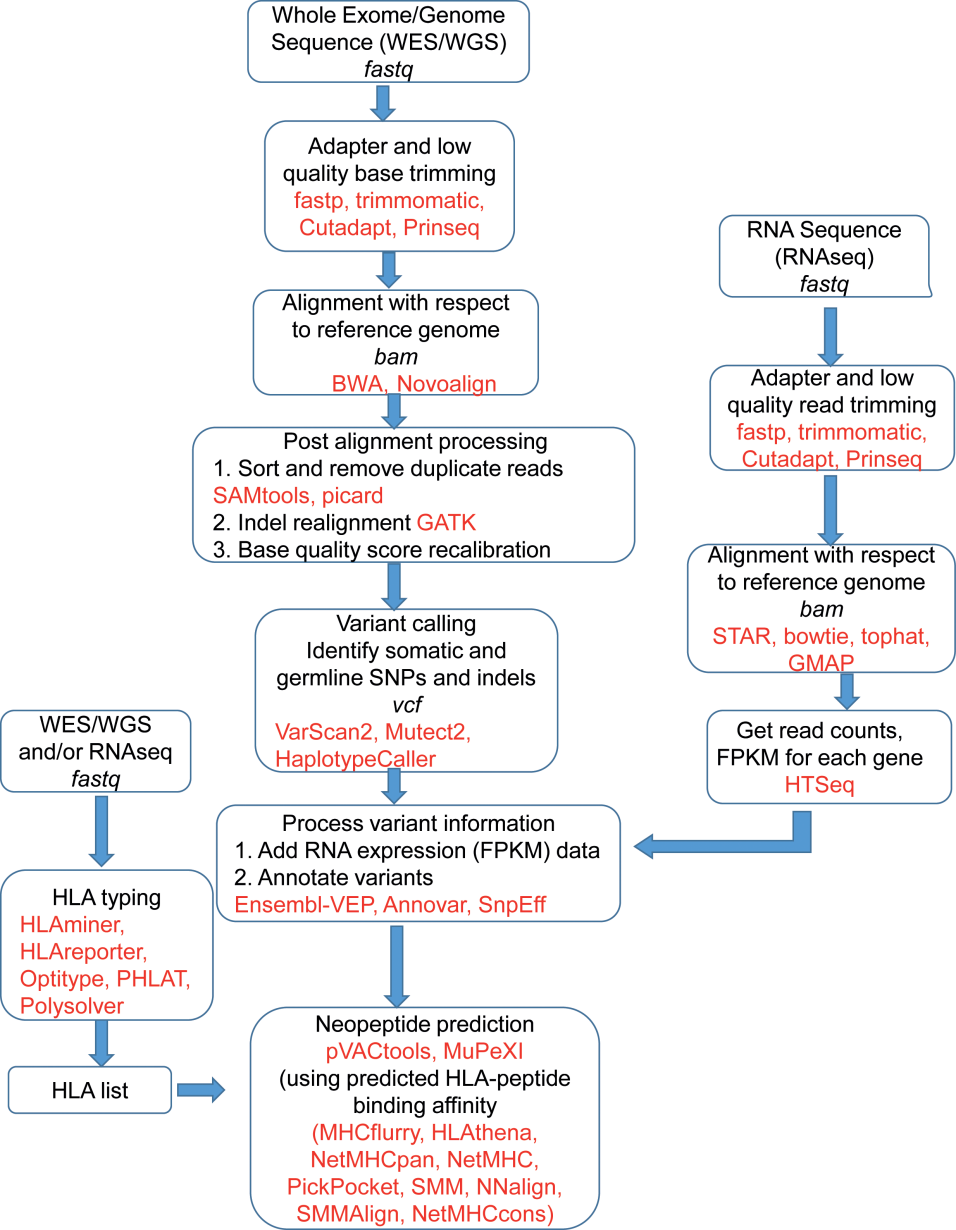
A schematic of the bioinformatics process of neoantigen vaccine design. Frequently used software and tools are in red. The file formats are in italics.

**Figure 2** shows the schematic of the bioinformatics process of vaccine design. The sequence reads, available in fastq file format, contain sequences and a quality score for each base representing the accuracy of the sequencer in identifying that base. The fastq files are pre-processed by trimming out low-quality bases and adapter sequences. Software like fastp (26), Trimmomatic (27), Cutadapt (28), and Prinseq (29) are used for trimming and FastQC (30) is very often used for quality checks. To identify tumor specific somatic nucleotide variants, both normal and tumor sequence reads are aligned or mapped to the reference human genome sequence assembly, available at NCBI and Ensembl (31) database. There are multiple aligner software available based on different algorithms. These algorithms include BWA, BWA-MEM (32), and Novoalign (33). The genome analysis tool kit (GATK) provides a bundle of software required for sequence analysis (34). Among different algorithms, BWA works for shorter sequences and BWA-MEM works for longer sequences. So for aligning WES, the BWA-MEM algorithm is preferred. mRNA sequence is also aligned in a similar fashion against the reference genome using specialized aligners. mRNAs are transcribed only from the exon parts of the genome by removing introns, but the reference genome contains both introns and exons. Hence, while aligning mRNA sequences, the splicing of exons should be taken care of. Among the mRNA sequence aligner software, STAR (35), GMAP (36), and Tophat2 (37) are splice-aware whereas Bowtie2 (38) is not splice-aware. For both WGS/WES and mRNA sequence alignment, the information is obtained in the form of a sequence alignment map (SAM) file or its binary counterpart BAM file. For mRNA sequences, expression count values of different mRNAs are extracted from the BAM file using software like HTSeq2 (39). For WES, the mapped sequences require post-processing, for which software like GATK, Picard (40), SAMtools (41) are often used. This post-processing includes the removal of duplicate reads, which originate from the same fragment of the DNA. Indel realignment is also recommended by realigning reads near detected indels to remove alignment artifacts. After the removal of duplicate reads, the base quality score recalibration (BQSR) is performed using GATK. In the BQSR process, using machine learning algorithms, the systematic errors made by the sequencer while calling the bases are estimated and base quality scores are calibrated accordingly. These recalibrated BAM files are further used for identifying different genetic variants.

The variant calling software identifies single nucleotide polymorphisms (SNPs) and small insertions and deletions (indels). The software includes VarScan2 (42), Mutect2 (43), HaplotypeCaller (44) and each of them provides output in a variant call format (vcf). The vcf files contain nucleotide mutations and other information like chromosome position and quality scores associated with variant detection. Since neoantigens are based on somatic mutations, germline variants are often excluded. However, software like pVACtools considers germline variants and other somatic variants which are proximal to the ‘somatic variant of interest’ for which neoantigen is being predicted (45). To identify the germline variants, the BAM file from a normal DNA sample is used. Also, some known variants are removed to isolate tumor specific variants. The known variants can be obtained from resources like the GATK resource bundle in a user friendly format (46) and also from dbSNP (47).

The vcf files containing variant information are further annotated which effectively tags the variants with other necessary information from different databases. This information includes gene information, transcript information, variant location, variant consequence (mutation type), and associated minor allele frequency (MAF), depending on the annotation and software used. The commonly used annotation software are Ensembl-VEP (48), ANNOVAR (49), SnpEff (50), and the databases are dbSNP (47), 1000 Genomes (51), etc. Since a vcf file may contain hundreds of mutations, the associated information is used for prioritizing the possible neopeptides.

These annotated vcf files are used for peptide prediction using software like pVACtools (21), MuPeXI (23). The prediction process is based on the binding with MHC molecules which is predicted by different software like MHCflurry (52), HLAthena (53), MixMHCpred (54), NetMHC (55), NetMHCpan (56), NetMHCcons (57), PickPocket (58), and SMM for MHC-I type whereas NetMHCIIPan (56), SMMAlign (59), and NNalign (60) are used for MHC-II molecules. Mei et al. observed that MixMHCpred 2.0.1, NetMHCpan 4.0, and NetMHCcons 1.1 perform well for predicting peptides binding to most of the HLA-I allomorphs (61). For a robust prediction of neopeptides, the list of relevant alleles, corresponding mRNA expression status of the mutated genes are required. The relevant alleles are predicted by HLA typing process which may be a clinical approach or analytical approach based on the WGS, WES or mRNA sequence data. The neopeptides, targeted for binding with MHC-I molecules are usually of 8-10 mer lengths whereas peptides targeted for binding with MHC-II molecules are usually longer, 13-25 mers (62). The neopeptide prediction software usually provides a large number of peptides that are further shortlisted based on the strength of peptide-MHC binding which is expressed in terms of IC50 values. IC50 < 50 nM is considered strong binding and IC50 >500 nM is considered non-binder (63). The mRNA expression strength and variant allele frequency are also considered for the final selection of peptides.

The peptides are further formulated following different strategies considering peptide solubility and stability. Oosting et al. developed a formulation that maintains stability for up to 32 weeks (64). The delivery strategy includes the use of mRNA vaccine (65), DNA vaccine (66), pulsed dendritic cells (67), and recombinant viruses (68). It also includes direct injection of unformulated vaccines (69). Different adjuvants like poly-ICLC, and helper peptides like tetanus are also used in the formulation. The administration process includes subcutaneous, intramuscular, and intravenous injections mostly in limbs.

### 2.3 Challenges

The neoantigen vaccine-based immunotherapy is a complex process involving several challenges (68). As the multistep design process involves the use of several computational tools, each of them containing an algorithm and each with advantages and disadvantages. This situation often results in wide variability in the output neopeptide sequences. For each analysis step, multiple software exists. The optimized combination of the software is required for building the pipeline for vaccine design. There is wide variability in identifying the mutations based on the WGS/WES data using different variant callers (70, 71). In case of high variance in mutations called by different variant callers, the consensus outputs can be considered as more reliable mutations (70–72). Similar high variability is observed in the process of HLA typing to identify MHC molecules from the sequence data (73, 74). Many HLA typing software exist, and they identify the HLA molecules in diverse ways using different computational and statistical approaches. The results of this software can be compared with the clinical HLA typing tests based on antigenic reactions, using the blood sample of patients. Here, the miscalled HLAs can be avoided for peptide binding. Also, for HLA class II typing, the number of HLA typing callers is less. The computational approach will identify a large number of neopeptides. Even, for a given mutation site, multiple peptides may be detected which may have varying lengths, and differ starting and ending positions of the sequence. Among them, the list of effective peptides need to be optimized based on criteria like HLA-peptide binding strength. Moreover, the neopeptide may not be effective if the corresponding HLA allele is deleted, not expressed, or epigenetically silenced as it reduces the possibility of its binding with the neoepitopes (75), so ideally these also should be verified.

There are also multiple biological implications. The binding is mediated between T cell exposed motifs (TCEM) of neopeptides with groove exposed motif HLA molecules. For better binding, and hence better T cell activation, Bremel et al. used peptides whose amino acids are altered maintaining TCEM core conservation (76). Tumors with a high mutational burden are more likely to have more number of neoantigenic peptides, which may lead to more neoepitope choices and better outcomes (77). The targeted somatic mutations should ideally be present in all cancer cells. These can be founder mutations that initiated cancer and thus possibly may be present in all lineage cells that form the bulk of cancer tissue. There may exist multiple subclonal mutations; consequently it may be better to target the dominant clone which may be present in the bulk of the cancer tissue. Selection of clonal and subclonal mutations can be achieved by establishing cancer cell content in the tissue used for sequencing and comparing mutant allele frequency with wild type/normal allele frequency. Also, the mutation may be heterozygous, present in one allele, the variant allele frequency should be preferably up to 50%, for the mutation to be considered for the vaccine target. In the case of homozygous mutation, the maximum allele frequency will approach 1.0. The designed peptide, synthesized *in vitro*, should be compatible with the physiological environment.

The neoepitopes can be formulated and administered in different vaccine formats (69), like mRNA vaccine (65), DNA vaccine (66), pulsed dendritic cells (67), and recombinant viruses (68). A proper choice is required. *In vitro* transcribed (IVT) mRNA vaccines have multiple advantages over other choices. As it does not integrate into the genome, the risk of insertional mutagenesis and infection is less (65). Apart from pulsed dendritic cells, B cells, macrophages, and splenocytes have also been tried which also act as adjuvants (78). Finally, the entire process should be cost-effective in terms of time, instrumental resources, and human resources.

## 3 Ongoing clinical trials

We noted that there are many clinical trials currently ongoing and can be accessed *via* the NIH ClinicalTrials website (https://www.clinicaltrials.gov/ct2/home). The trials which involve the administration of vaccines on human subjects are listed and tabulated in **Table 1**. We observed that trials mostly involve multiple types of cancers, and are also dedicated to specific sites like pancreatic cancer and breast cancer. We provide a brief review of different cancer types covered by neoepitope/neoantigen clinical trials.

**Table 1:** List of clinical trials using neoantigen vaccine therapy. Data accessed on December 20, 2022.

NCT Number	Conditions	Intervention/Drug	Formulation	Administration	Sponsor/Collaborators
NCT03122106	Pancreatic Cancer	Biological: Personalized neoantigen DNA vaccine	Neoantigen DNA vaccine	Intramuscular injections using TDS-IM system	Washington University School of Medicine|National Cancer Institute (NCI)
NCT03956056	Pancreatic Cancer	Biological: Neoantigen Peptide Vaccine|Drug: Poly ICLC	Peptides with poly-ICLC	Subcutaneous injection to limb	Washington University School of Medicine|National Institutes of Health (NIH)|National Cancer Institute (NCI)
NCT03645148	Pancreatic Cancer	Biological: iNeo-Vac-P01	iNeo-Vac-P01 (5 – 20 peptides) vaccine with GM-CSF adjuvant	Subcutaneous injections at the dose of 100 μg per peptide	Zhejiang Provincial People's Hospital|Hangzhou Neoantigen Therapeutics Co., Ltd.
NCT04161755	Pancreatic Cancer	Drug: Atezolizumab, mFOLFIRINOX |Biological: RO7198457	RO7198457	Not available	Memorial Sloan Kettering Cancer Center|Genentech, Inc.
NCT04105582	Breast Cancer|Triple Negative Breast Cancer	Biological: Neo-antigen pulsed dendritic cell	Neo-antigen pulsed autologous dendritic cell	Not mentioned	Universidad Nacional de Colombia|FundaciÃ³n Salud de los Andes
NCT04879888	Breast Cancer Female	Biological: Peptide pulsed Dendritic cell	Peptide-pulsed autologous dendritic cells	Intradermal vaccination	Universidad Nacional de Colombia|FundaciÃ³n Salud de los Andes|Instituto Colombiano para el Desarrollo de la Ciencia y la TecnologÃa (COLCIENCIAS)|Subred Integrada de Servicios de Salud Sur ESE - Colombia (South America)
NCT03199040	Triple Negative Breast Cancer	Drug: Durvalumab|Biological: Neoantigen DNA vaccine|	neoantigen DNA vaccine with durvalumab	Two injections using TDS-IM system	Washington University School of Medicine |MedImmune LLC|National Cancer Institute (NCI) |National Institutes of Health (NIH)
NCT02348320	Triple Negative Breast Cancer	Personalized polyepitope DNA vaccine	Naked plasmid DNA vaccine	Intramuscularly using a TriGrid electroporation device	Washington University School of Medicine Susan G. Komen Breast Cancer Foundation
NCT03715985	Melanoma,Non Small Cell Lung Cancer, Bladder Urothelial Cancer	Drug: EVAX-01-CAF09b	Up to 15 peptides with CAF09b as adjuvant.	Intraperitoneal and intramuscular injections	Herlev Hospital
NCT03673020	Solid Tumor, Adult	Biological: ASV® AGEN2017 + QS-21 StimulonÂ® adjuvant	ASV® AGEN2017 with QS-21 Stimulon® adjuvant	Subcutaneous injection	Agenus Inc.
NCT02992977	Advanced Cancer	Biological: AutoSynVax vaccine	AutoSynVax™ vaccine with QS-21 Stimulon® adjuvant	Subcutaneous injection	Agenus Inc.
NCT04509167	Neoplasms	Biological: Neoantigen Peptides	Multi-peptide vaccine with adjuvant Montanide ISA-51 VG	Intradermal injection	Instituto de Medicina Regenerativa
NCT03480152	Melanoma|Colon Cancer|Gastrointestinal Cancer|Genitourinary Cancer|Hepatocellular Cancer	Biological: Personalized Cancer Vaccine	Up to 15 peptides using mRNA based vaccine	Intramuscular injection	National Cancer Institute (NCI)|National Institutes of Health Clinical Center (CC)
NCT03568058	Advanced Cancer	Drug: personalized vaccine|Drug: Pembrolizumab	Personalized vaccine	Intravenous infusion	Ezra Cohen|University of California, San Diego
NCT03633110	Cutaneous Melanoma|Non-small Cell Lung Cancer|Head and Neck cancer|Urothelial Cancer|Renal Cell Cancer	Biological: GEN-009 Adjuvanted Vaccine|Drug: Nivolumab, Pembrolizumab	Peptides with poly-ICLC	Subcutaneous injection	Genocea Biosciences, Inc.
NCT03639714	Non Small Cell Lung Cancer|Colorectal Cancer|Gastroesophageal Adenocarcinoma|Urothelial Carcinoma	Biological: GRT-C901, GRT-R902, nivolumab, ipilimumab	20 peptides each having 25 amino acids arranged in a cassette with helper epitopes PADRE and tetanus toxoid. Virus vaccines as vector	Intramuscular injection	Gritstone bio, Inc.|Bristol-Myers Squibb
NCT03662815	Advanced Malignant Solid Tumor	Biological: iNeo-Vac-P01	iNeo-Vac-P01 (5 – 20 peptides) vaccine with GM-CSF adjuvant	Subcutaneous injections at the dose of 100 μg per peptide	Sir Run Run Shaw Hospital|Hangzhou Neoantigen Therapeutics Co., Ltd.
NCT03300843	Melanoma| Gastrointestinal Cancer| Breast Cancer| Ovarian Cancer| Pancreatic Cancer	Biological: Peptide loded dendritic cell vaccine	Autologous mature dendritic cells loaded with long peptides and minimal epitopes	Intravenous and subcutaneous injections	National Cancer Institute (NCI)|National Institutes of Health Clinical Center (CC)
NCT03548467	Locally Advanced or Metastatic Solid Tumours	Biological: VB10.NEO Drug: Bempegaldesleukin	VB10.NEO in with bempegaldesleukin (NKTR-214)	Intravenous injection	Nykode Therapeutics ASA|Nektar Therapeutics|Vaccibody AS
NCT03359239	Urothelial/Bladder Cancer	Drug: Atezolizumab, Poly ICLC |Biological: PGV001	Up to 10 peptides, one teatanus helper peptide mixed with poly-ICLC.	Intravenous infusion	Matthew Galsky|Genentech, Inc.|Icahn School of Medicine at Mount Sinai
NCT03532217	Prostate Cancer	Drug: Nivolumab, Ipilimumab |Biological::Neoantigen DNA vaccine	Engineered replication-competent vaccinia and Fowlpox virus	Two intramuscular injections using a TriGrid electroporation device	Washington University School of Medicine|Bristol-Myers Squibb|Prostate Cancer Foundation|The Foundation for Barnes-Jewish Hospital|Bavarian Nordic
NCT01970358	Melanoma	Biological: Poly-ICLC, Peptides	Peptides with poly-ICLC	Subcutaneous injection	Dana-Farber Cancer Institute
NCT05309421	Melanoma Stage IVMelanoma Stage III	Drug: EVX-01Drug: Pembrolizumab 25 MG/ML	EVX-01 vaccine	Intramuscular injection	Evaxion Biotech A/S|Merck Sharp & Dohme LLC
NCT04455503	Melanoma Stage IVMelanoma Stage III	Drug: EVX-02A|Drug: EVX-02B|Drug: EVX-02A OR EVX-02B	EVX-02A or EVX-02B vaccine	Intramuscular injection	Evaxion Biotech A/S| Novotech (Australia) Pty Limited
NCT03422094	Glioblastoma	Biological: NeoVax, Nivolumab, Ipilimumab	Up to 20 peptides with poly-ICLC	Subcutaneous injection	Washington University School of Medicine|Bristol-Myers Squibb
NCT02510950	Glioblastoma	Biological: Personalized peptide vaccine|Drug: Poly-ICLC, Temozolomide	Peptide vaccine with poly-ICLC	Not available	Washington University School of Medicine

### 3.1 Pancreatic cancer

The ongoing phase 1 clinical trial NCT03122106 of the neoantigen DNA vaccine against pancreatic cancer addresses its safety and immunogenicity in patients with adjuvant chemotherapy and surgical resection. The hypothesis is to determine if this neoantigen DNA vaccine is capable of developing CD4+ and CD8+ T cell responses. These vaccines are comprised of prioritized neoantigens together with personalized mesothelin epitopes (79). The clinical trial NCT03956056 is also targeted toward pancreatic cancer patients to evaluate immune cell responses to neoantigen vaccines co-administered with immunostimulant poly-ICLC. Additionally, the clinical trial NCT03645148 is dedicated to pancreatic cancer patients of Chinese origin with a low mutational burden. The vaccine iNeo-Vac-P01 was developed utilizing their in-house pipeline iNeo-Suite. The vaccine contained up to twenty peptides. It was administered to patients having low mutational burden and appeared safe with enhanced effector T cell counts (80). The response of the vaccines also depends on the adjuvant drugs. The ongoing trial NCT04161755 uses the drug atezolizumab along with mFOLFIRINOX in the context of pancreatic cancer patients undergoing neoantigen vaccine therapy.

### 3.2 Breast cancer

Neoantigen vaccine therapy is being tried in breast cancer patients; specifically, triple negative breast cancers (TNBC) where genetic instability is associated with a high mutational burden. In the clinical trial NCT04105582, up to 25 neopeptides are going to be administered by the autologous dendritic cells over a 16 week span. Another clinical trial NCT04879888 also uses peptide pulsed autologous dendritic cells at six doses on nine TNBC patients. The clinical trial NCT03199040 is designed to evaluate the response of neoantigen vaccines in the presence and absence of the drug durvalumab in triple negative breast cancer patients. The clinical trial, identified as NCT02348320, is an ongoing phase 1 trial of a polyepitope DNA vaccine against triple negative breast cancer. The immunogenicity and safety of the vaccine are being evaluated in the trial.

### 3.3 Pan-cancer

In a pan-cancer study, researchers are looking for the effects of the EVAX-01-CAF09b vaccine in the metastatic condition of malignant melanoma, NSCLC, and bladder urothelial cancer. The vaccine will be derived using the PIONEER platform and will contain 5-15 peptides (NCT03715985). Agenus Inc. conducted multiple trials on the safety and tolerability of their ASV^®^ AGEN2017 with QS-21 Stimulon^®^ Adjuvant in solid tumors but their enrolled patients were limited to three only (NCT03673020, NCT02992977). The clinical trial NCT04509167 uses Montanide ISA-51 VG as an adjuvant along with 0.5mg of each predicted peptide. The clinical trial NCT03480152 on 4 patients having metastatic melanoma and colon cancer observed enhanced T cell response with no objective response in all patients (81). The anti-PDL1 antibody drug pembrolizumab is being assessed in the neoantigen vaccine trial NCT03568058 in NSCLC, head and neck squamous cell carcinoma (HNSCC), classical Hodgkin lymphoma (cHL) and other solid tumors. This study will observe the immune response when pembrolizumab is administered six weeks before vaccination, at the time of vaccination, and after vaccination. Genocea Biosciences is also conducting a clinical trial (NCT03633110) on 24 participants having different cancers. This trial also uses the drug pembrolizumab along with nivolumab to evaluate the efficacy of vaccine therapy.

A clinical trial with NCT number NCT03639714 assigned to the company Gritstone bio is evaluating the early clinical activity, dose, immunogenicity, and safety of a personalized neoantigen cancer vaccine GRT-C901 and GRT-R902 integrated with the drugs nivolumab and ipilimumab for NSCLC, microsatellite stable colorectal cancer, gastroesophageal adenocarcinoma, and metastatic urothelial cancer patients. The primary objective is to look for any adverse events, serious adverse events (SAEs), and dose-limiting toxicities (DLTs). As well, their objective is to compute Objective Response Rate (ORR) in Phase 2 and identify the recommended Phase 2 dose. Their interim results demonstrate an enhanced overall survival period (82). Gritstone bio is also conducting another clinical trial (NCT03794128) to explore the personalization aspect of neoantigen vaccines. Their objective is to identify personalized and shared vaccines in the context of different cancers involving 93 patients. NCT03662815 refers to a trial on Chinese patients with solid tumors. The outcome shows that of 30 patients, 20 had no adverse effects and 80% of peptides enhanced immune response (83). NCT3300843 was also initiated for pan-cancer study using peptide loaded dendritic cell vaccines but was terminated due to low accrual. Individualized VB10.NEO vaccine and bempegaldesleukin (NKTR-214) are being used in the clinical trial NCT03548467 for patients at the metastatic stage. It plans for 14 vaccinations for each of the 65 patients and bempegaldesleukin (NKTR-214) will be given after at least four doses of vaccinations. The primary goal is to measure the safety and adverse effects of the vaccine. Secondary outcome measurement includes measuring immunogenicity by T cell activity to each neoepitope, ORR, duration of response, progression free survival, and survival at the end of treatment.

### 3.4 Other cancers

Similar to the trial NCT04161755, clinical trial NCT03359239 aims to determine the effects of atezolizumab in combination with a personalized cancer vaccine, PGV001 (84) for locally advanced or metastatic urothelial cancer patients. A clinical trial is also evaluating the immune response of a shared antigen vaccine PROSTVAC and tumor specific antigens generated DNA vaccine with nivolumab (anti-PD-1), and ipilimumab (anti-CTLA-4) for checkpoint blockade (NCT03532217). The ongoing open label phase 1a/1b clinical trial (NCT03970382) is focusing to evaluate the efficacy, feasibility, and safety of NeoTCR-P1 T cells in subjects with metastatic hormone-sensitive prostate cancer. NCT03040791 is another trial involving pancreatic cancer patients which also utilizes nivolumab to explore DNA repair defects (DRD), mainly in the Homologous Recombination (HR) pathway. The effect of nivolumab is also being investigated with or without ipilimumab in female patients suffering epithelial ovarian, primary peritoneal, or fallopian tube cancer in clinical trial NCT02498600. The outcomes will be measured as per response evaluation criteria in solid tumors, survival periods, and incidence of adverse events in advanced stages of the disease.

Trials are being conducted on skin cancer melanoma which is also characterized by patient specific mutation. Clinical trial with NCT number NCT01970358 enrolled 20 melanoma patients to whom peptide vaccine NeoVax targeting up to twenty peptides was administered starting from day 1 to 162 along with poly-ICLC. It resulted in induced T cell response sustaining over years (85, 86). A clinical trial NCT05309421 is designed to determine the efficacy of EVX-01 vaccine on advanced melanoma patients. The trial will evaluate whether checkpoint inhibitor therapy using pembrolizumab works better when utilized in conjunction with EVX-01 vaccine (87). Clinical trial NCT04455503 also treats advanced melanoma patients but with two types of EVX-02 vaccines with nivolumab in two cohorts. Depending on the study results the third cohort will receive either one of the two types of EVX-02 vaccine. This study will measure safety and tolerability by measuring vital signs like heart rate, blood pressure, and physical examination. Neoepitope-specific T cells will be monitored by ELISPOT. Other pharmacodynamic responses of EVX-02 will be assessed by MHC I multimer analyses detecting neoepitope-recognizing CD8+ T cells and by flow cytometry to detect vaccine induced intracellular cytokine response. Relapse free survival period will be measured as secondary outcomes. A trial (NCT03422094) based on neoantigen vaccine therapy on glioblastoma patients was initiated but later focus was changed to cell therapy. Clinical trial NCT02510950 targeting glioblastoma patients did not proceed due to financial limitations.

We observed variations in the vaccine formulation and administration strategies followed by different trials. The number of chosen peptides varied from 5 - 20 depending on the mutational burden. These peptides are often applied with adjuvants. Poly-ICLC is used as an adjuvant in multiple trials. Poly-ICLC stimulates the release of cytokines and the production of interferon-gamma. The administration process and doses also vary. Intravenous, intramuscular, and subcutaneous injections at limb organs are used for administration. The dose typically remains around 100μg per peptide. The treatment typically continues for several months, depending on its effects. Table 1 lists different formulation and administration strategies observed in the trials.

## 4 Outcomes

Apart from the ongoing trials, several clinical trials already published their outcomes. In this section, we discuss those outcomes. Mismatch repair (MMR) deficient cells often lead to cancers due to the accumulation of numerous unrepaired mutations like base mismatches, insertions and deletions. This accumulation of mutations may affect cell cycle control genes and promote cancer growth. In this regard, Ott et al. have conducted several studies. In a study conducted on six melanoma patients, the clinicians used up to 20 neoantigens in each patient. They observed no recurrence in 25 months for four patients and for two patients, vaccination followed by anti-PD-1 therapy resulted in complete regression (88). They reported similar observations in glioblastoma patients also (89). Ott et al. also reported a neoantigen-based vaccine NEO-PV-01 along with PD-1 blockade in melanoma, NSCLC or bladder cancer patients. The vaccine showed CD4+ and CD8+ T cell response post vaccination with cytotoxic phenotype which could move to the tumor and mediate the killing of tumor cells. The treatment was found to be safe and no adverse events were reported (NCT02897765) (90). A single mRNA vaccine was presented by Cafri et al. to treat gastrointestinal cancer patients. It was developed by using lymphocytes that infiltrated tumors to detect immunogenic mutations that were expressed in the tumors of the patients. The vaccine (NCT03480152) was found to be safe and generated T cell responses targeting KRAS-G12D mutation. It also exhibited potential to develop vaccines integrated with checkpoint inhibitors or adaptive T cell therapy for common epithelial cancers (81).

Dendritic cells (DC) are often used for administering neoantigens. Carreno et al. vaccinated three melanoma patients with dendritic cell based vaccines and observed enhanced response of T cells (91). Ding et al. also used peptide-pulsed autologous DC vaccine for conducting a clinical trial involving twelve advanced lung cancer patients. They administered 12 – 30 peptides in doses ranging 3 – 14 doses per person. However, the median progression-free survival was limited to 5.5 months (92). In another study, rather than using a set of peptides, researchers used a single peptide targeting only IDH1 mutation in glioma patients (93). Instead of personalized peptides, Mueller et al. used ‘shared neoantigen’, specific to H3.3K27M mutation among nineteen glioma patients and it was well tolerated with median overall survival of 16.1 months (94). Hilf et al. vaccinated newly diagnosed glioma patients with unmuted antigens first and then with targeted neoepitopes. Unmutated antigens evoked sustained responses of central memory CD8+ T cells and neoepitopes helped to develop CD4+ T cell responses. This combination therapy showed strong immunogenicity (95).

Neoantigen vaccine was tested on ten hepatocellular carcinoma (HCC) patients, and showed no adverse effects with a median recurrence free survival period 7.4 months (96). Kloor et al. performed phase 1 and 2 clinical trials (Micoryx) to evaluate frameshift peptide (FSP) based neoantigen vaccines. This trial is highly relevant in that it demonstrates the possibility of an effective cancer-preventive vaccine which may work among high-risk populations. They selected patients who have completed their chemotherapy and colorectal cancer (stage III or IV) with MMR deficiency. The trial consisted of four subcutaneous vaccination cycles admixed with Montanide ISA-51 VG. Phase I focused on the safety and toxicity of the vaccines, whereas phase II evaluated the cellular and humoral immune response. The results showed humoral and immune responses in all of the patients. Grade 2 injection site reactions were observed in three patients, but no adverse events occurred. Hence, FSP neoantigen based vaccination was observed to be well tolerated with good immune response and may emerge as a promising cancer preventive as well as a treatment for MMR-deficient cancers (97). Kristensen et al. found that only 1.8% of all neopeptides are present within tumor-infiltrating lymphocytes (TILs) infusion products in melanoma. They validated that the presence of neoepitope-specific CD8+ T cells helps in better survival (98). Although an *ex vivo* study but worth mentioning, in the case of breast cancer cells, the co-culture of neoantigen-pulsed DCs and lymphocytes successfully induced cytotoxic T lymphocytes (CTLs) response against cancer cells (99). Holm et al. treated metastatic urothelial cancer patients with peptides derived from exome sequence data and observed an increase in T cell response after 3 weeks of treatment which also facilitated the activity of immune checkpoint inhibitors (100). Miller et al. correlated somatic mutation and neoantigen burden with survival time from data collected in a clinical trial on 664 myeloma patients. Two-years progression free survival rate reduces from 0.726 to 0.493 and from 0.729 to 0.555 for high somatic mutation and neoantigen burden respectively (86). Palmer et al. reported the interim result of a clinical trial that uses a combinatory approach in colorectal cancer. They have used heterologous chimpanzee adenovirus (ChAd68) and self-amplifying mRNA(samRNA)-based neoantigen vaccine in combination with immune checkpoint inhibitor drugs nivolumab and ipilimumab; and they observed a median OS 8.7 months (82). A comparative study between patients treated with neoantigen specific T cells and anti PD-1 molecules and patients treated with only anti PD-1 molecules revealed patients treated with neoantigen specific T cells have better progression free survival time (13.8 and 4.2 months). However, the overall survival period was the same (101). In a phase 1b study on three pancreatic ductal adenocarcinoma (PDAC) patients, a combination of chemotherapy, dendritic cells with neopeptides and anti PD-1 drug nivolumab was used to enhance the efficacy of the vaccine (102). Clinical trial NCT03645148 reported the outcome observed on seven advanced pancreatic cancer patients. Using the vaccine iNeo-Vac-P01 the mean overall survival period reached 24.1 months whereas progression free survival period was 3.1 months (80). In a case study on a 62 year old pancreatic cancer patient, Sonntag et al. used four peptides derived from two mutations. The vaccination started along with chemotherapy, then chemotherapy stopped, and monthly doses of vaccines continued. The patient had four years of progression free survival, at the time the report was published (103). The clinical trial NCT04688385 published a report on the effect of multi-peptide vaccine on leukemia patients. It developed a workflow for off-the-shelf peptide warehouses which can be applicable for broad personalized therapeutics (104). Overall, we observe that the clinical trials employing neoantigens are showing promising results in terms of immunogenicity and safety. However, on-time delivery of these personalized vaccines to patients remains a challenge.

## 5 Outlook

Based on our literature review, promising outcomes are observed in the published neoantigen vaccine trials. Neoantigen vaccines are enhancing T cell responses while mitigating other side effects. However, the application is still limited to cases of high mutational load. This limitation can be optimized through rational design. We need a better understanding on the molecular mechanism of the neopeptides. Additionally, neopeptides targeting MHC class II type should be explored to enhance CD4+ T cell responses. Apart from IEDB, a few databases have also been developed that catalog neopeptides that, thus far, have been detected and utilized in preclinical and/or clinical environment. The NeoPeptide database contains characteristics of neoantigens reported in the literature and immunological resources (105). The Cancer Immunome Atlas (TCIA) provides results obtained primarily from TCGA (106). The Cancer Antigenic Peptide Database (CAPED) contains information on peptides, mutations, and associated HLA molecules (107). Tumor-Specific NeoAntigen database (TSNAdb) (108), Cancer Epitope Database and Analysis Resource (CEDAR) (109), and NEPdb (3) are also available. These databases help to find neopeptides whenever a mutation is detected.

We have observed neoantigen vaccines are accompanied by different adjuvant drugs. Among the adjuvant drugs, immune checkpoint blockade drugs are widely used. Drugs like nivolumab, ipilimumab and pembrolizumab are used in multiple types of cancers. Cancer cells express PD-L1 on their surface which binds to PD1 which is present on the surface of the T cells, this results in the inactivation of T cell and the lack of immune response of T cell against cancer cells. Nivolumab blocks PD-L1 binding with PD-1 which results in T cells retaining their immune activity and initiates an immune response against the cancer cells. These active T cells enhance the effectiveness of the treatment. Pembrolizumab also targets PD-1. Ipilimumab targets cytotoxic T-lymphocyte-associated protein 4 (CTLA-4) (110). The combination of nivolumab and ipilimumab is also used (111). Hence, the proper selection of adjuvant drugs at appropriate doses and times plays a crucial role in the success of neoantigen immunotherapy.

We observe that the neoantigen vaccines appear safe with limited side effects. However, the survival period is still not promising. This may be a result of the majority of the trials currently ongoing are conducted on patients who have already reached the metastatic stage or the late stage of the disease. An early intervention with neoantigen vaccines may provide a longer survival period for the patients. It needs to be validated by clinical trials in the future.

The vaccine administration process including the peptide carriers also needs to be more streamlined. Currently, mRNA vaccine, DNA vaccine and pulsed dendritic cells are mostly used as carriers. Compared to TAAs, neoantigens show stronger immunogenicity and binding towards HLAs are not affected by central immunological tolerance (5). Neoantigens are resultant of mutations in tumor cells during tumorigenesis. The mutation landscape also evolves continuously during tumorigenesis and disease progression (5). It makes neoantigens specific to the tumor stage and more trials are needed for exploring patients of different stages. As mentioned in the introductions section and based on the NIH clinical trial website, we noted several clinical trials that are about to initiated. The results from those studies will provide a better landscape on the therapeutic efficacy of neoantigen immunotherapy.

## 6 Conclusions

Based on the existing circumstances, we conclude neoantigen vaccines are capable of exhibiting tumor-specific immunogenicity in different types of solid tumors. They leverage CD4+ and CD8+ effector T cells across cancer types. However, there is an enormous requirement for improvements in several aspects like the optimized design of neoantigens to ensure the efficacy of the vaccine. Conducting *ex vivo* studies on the effect of peptides on tumor cells collected from patients will be helpful for a well-defined vaccine design. Further studies are required to evaluate the possibility of the existence of patient subtypes based on the responses to neopeptides. If corroborated, it will make the vaccine production process more economical both in terms of money and time. Researchers and clinicians should explore the possibility of applying vaccines to patients at the earlier stages of the disease which may provide a longer survival period. We are looking forward to improved treatment options for cancer patients.

## Author contributions

NB and SC wrote the manuscript. All co-authors have read, revised as required, and agreed with the content of the manuscript.
